# Microbiome functional gene pathways are indicative of cognitive performance in older adults at risk for Alzheimer's disease

**DOI:** 10.1080/19490976.2026.2676162

**Published:** 2026-05-24

**Authors:** Abigail L. Zeamer, YuShuan Lai, Ethan Loew, Victoria Sanborn, Matthew Tracy, Cynthia Jo, Danielle Ferdinand, Doyle V. Ward, Shakti K. Bhattarai, Johnathan Drake, Beth A. McCormick, Vanni Bucci, John P. Haran

**Affiliations:** a Department of Microbiology, University of Massachusetts Chan Medical School, Worcester, MA, USA; b Program in Microbiome Dynamics, University of Massachusetts Chan Medical School, Worcester, MA, USA; c Rhode Island Hospital, Providence, Rhode Island, USA; d Department of Emergency Medicine, University of Massachusetts Chan Medical School, Worcester, MA, USA

**Keywords:** Alzheimer's disease, mild cognitive impairment, cognition, ADAS-Cog, clinical dementia rating (CDR) scale, NIH toolbox, memory, executive function, microbiome, methionine, putrescine, polyamines, cysteine, urea cycle, folate, vitamin B12

## Abstract

Disturbances in the gut microbiome are increasingly correlated with neurodegenerative disorders, including Alzheimer's disease. Multiple lines of emerging evidence are consistent with the microbiome's involvement in disease pathology in AD by triggering or potentiating systemic and neuroinflammation, thereby influencing disease pathology through the “microbiota–gut–brain axis.” Currently, the copathologies contributing to cognitive decline and symptomatic progression in AD remain unknown and understudied. Changes in the gut microbiome composition may offer clues to potential systemic physiologic and neuropathologic changes that contribute to cognitive decline. Here, we recruited a cohort of 260 older adults (aged 60 y or older) living in the community and followed them over time, tracking objective measures of cognition, clinical information, and gut microbiome samples. Subjects were classified as healthy controls, exhibiting mild cognitive impairment, or having dementia based on clinical assessments. Using metagenomic sequencing and gene pathway analyses, we found that certain microbial-encoded metabolic pathways correlated with worse cognitive performance. Specifically, genes involved in the urea cycle, polyamine synthesis, or the metabolism of methionine and cysteine predicted worse cognitive performance. Our study suggests that the gut microbiome composition may be linked to cognitive impairment along the AD continuum and points to microbial metabolic pathways that may potentiate disease.

## Introduction

Alzheimer's disease (AD) is a highly prevalent and progressive neurodegenerative disease and the most common cause of dementia in older adults with no known prevention or cure.^1^ AD pathology is characterized by beta-amyloid plaques, tau-based aggregates that form neurofibrillary tangles (NFTs), neuroinflammation, and overall gross neuronal atrophy. In individuals with AD, symptoms often appear years after changes in the brain have begun.^2-4^ The diagnosis of AD has advanced from post-mortem diagnosis to symptomatology-based and now includes the use of imaging and laboratory-based biomarkers.^3^ ^,^ ^5-7^ The latest recommendations by the Alzheimer's Association Workgroup, released in 2024, now include staging based on biologic markers and clinical symptoms.^8^ Despite recent success with the development of the first disease-modifying therapies for AD,^9^ ^,^ ^10^ it is currently understood that there are a number of other potentially targetable mechanisms contributing to AD pathologic change, which warrant exploration for other avenues of intervention.

The microbiota-gut-brain (MGB) axis describes the bi-directional communication between gut microbes and the central nervous system through cytokines, hormones, neuronal signals, and bacteria-derived molecules.^11-13^ Dysbiosis or disruptions of microbiome composition and diversity have been associated with multiple neurodegenerative conditions that also have a strong neuroinflammatory component, including Multiple Sclerosis, Parkinson's Disease, and AD.^14-16^ In 2017, Vogt and colleagues described the first evidence of a direct link between dysbiosis of the gut microbiome and AD in humans using 16S sequencing,^17^ and subsequently multiple other groups have published compelling evidence of this link in germ-free and AD mouse models.^18-21^ Since then, there has been growing literature and interest in studying the gut microbiome's role in AD pathogenesis. Numerous studies have identified differences between taxa with general trends suggesting that AD is associated with pro-inflammatory taxa.^19-21^ However, these studies remain correlative, and the results have been rather heterogeneous between groups in terms of which individual taxa correlate with AD and directionality of correlations. This is not uncommon in microbiome research and is likely the result of different cohort populations, cohort sizes, different methods of clinical staging or grouping, as well as sequencing methods and instruments used. What has emerged as a more consistent pattern is the finding that specific microbially derived metabolites are associated with AD.^19-25^ We believe that metabolic pathway analysis might hold more clues to the role of the microbiome; examining metabolic pathways and metabolites directly inherently incorporates the effects of microbial community structures, which may have synergistic or antagonistic effects beyond individual species. Furthermore, examining metabolites may offer avenues toward understanding specific mechanisms in AD pathogenesis, as the field increasingly recognizes the role of systemic inflammation and loss of barrier integrity at both the gut and the blood–brain barriers. For instance, butyrate and secondary bile acids, both derived from gut microbial metabolism, have been associated with local and systemic signaling as well as modulating immune activity.^19^ ^,^ ^24^ ^,^ ^26^ The specific mechanisms by which microbial metabolites and gut dysbiosis contribute to AD pathology are beginning to emerge, likely involving a complex interplay between the gut microbiome, its influence on immune activation, neuroinflammation, and ultimately neuronal damage. To fully dissect the influence of the MGB axis in disease progression, microbiome studies must now include more objective clinical measures, both biologic and symptomatic, across the continuum of healthy individuals to those with later-stage AD.

To help bridge this gap, our study here focuses on clinical measures of cognition. There are currently few studies that examine objective, standardized tests of cognition in older adults in conjunction with microbiome analysis.^27^ In the context of AD, a cross-sectional study of women found associations between microbial taxa and functional gene pathways with cognitive tests,^28^ while an intriguing case report found improvement in multiple standardized cognitive tests in a 90-y-old patient with documented AD after fecal microbiota transplant for treatment of *Clostridioides difficile* infection.^29^ Progress or stage of AD is commonly measured using mental status scales such as the Mini-Mental State Examination (MMSE) and clinical dementia rating (CDR).^30^ ^,^ ^31^ Another test is the Alzheimer's Disease Assessment Scale Cognitive subscale 13-item (ADAS-Cog-13), currently favored in clinical trials and longitudinal studies due to its high sensitivity and ability to track AD-specific progression.^32^ The ADAS-Cog-13 is a global cognitive measure attained by the combination of 13 tasks covering the cognitive domains of memory, language, praxis, and executive functioning.^33^ It has also been shown to be more sensitive for detecting early/milder cases of cognitive dysfunction.^34^ The NIH has also developed a Cognitive Battery (CB) as part of their Health Toolbox for the Assessment of Neurologic and Behavioral Function. Although not developed specifically for the older adult population or dementia disorders, the Toolbox assessments are easy to administer, standardized tests that offer sensitive measures of specific cognitive domains such as executive function, attention, and memory.^35-37^ We recently showed that components of the NIH toolbox can be administered effectively to assess specific cognitive functions in patients with AD and that specific tested functions predicted global cognitive decline.^38^ We believe that combinations of the above tests will serve the field in following disease progression and compare cognitive measures objectively across different research studies.

Here, we utilize the Gut–Brain Alzheimer's Disease Inflammation and Neurocognitive Study (GAINS)^39^ to explore the microbiome of older individuals across a range of cognitive states using the ADAS-Cog-13 and the NIH Toolbox CB. Within this cohort, we found that the gut microbiome composition correlated well with these objective measures of cognition in both the MCI and AD groups. Analysis of microbial-encoded gene pathways also points to dysbiotic signatures in which the selection of microbial metabolites was correlated with worse cognitive performance. Our study adds to the growing literature implicating specific microbial metabolites in the systemic and functional outcomes of AD.

## Materials and methods

### Study design and subject categorization

Older individuals (60 y or older) residing independently in Massachusetts were recruited to the Gut-brain Alzheimer's Disease Inflammation and Neurocognitive Study (GAINS). Individuals with AD or MCI (see grouping criteria below) were targeted along with healthy controls, usually a family member of a subject with AD. We excluded participants with antibiotic use in the preceding 60 d and those with gastrointestinal conditions (i.e., bowel shortening surgery or colostomy), which are likely to grossly affect gastrointestinal physiology. We also excluded participants with other neurodegenerative diagnoses, such as Lewy body disease and Huntington's disease. During enrollment, demographics (i.e., age, sex, race, and education) and medical history were collected. At enrollment and each follow-up visit (occurring every 90 d), fecal samples, nutritional status (using the Mini-Nutritional Assessment,^40^), frailty (measured using the Canadian Study of Health and Aging's 7-point Clinical Frailty Scale (CFS)),^41^ hospital exposure and medications were collected. We also performed a battery of cognitive assessments at each visit, including the Alzheimer's Disease Assessment Scale-Cognitive Subscale 13 (ADAS-Cog-13),^33^ the Clinical Dementia Rating Scale (CDR),^39^ and the Cognitive Battery (CB) of the National Institutes of Health (NIH) Toolbox for the assessment of neurological and behavioral function.^42^ ^,^ ^43^

Subjects were categorized into three groupings post hoc: Alzheimer's disease (AD), mildly cognitively impaired (MCI), and cognitively normal healthy controls (HC). For subjects to be labeled as having AD, they or their caregiver self-reported a diagnosis by a clinician. To confirm the clinical diagnosis, research staff reviewed medical records, including neurological assessments, neuroimaging, and other tests. The current standard of care and clinical guidelines remain largely based on symptomology, supplemented by neuroimaging and CSF analyses where available. Blood-based biomarkers are now available to some institutions, but laboratory value thresholds for diagnosis vary across different institutions. We note that these supplemental tests are often not ordered if the results would not alter the clinical management of patients. For the purposes of this study, AD was a clinical diagnosis given the lack of uniformity of confirmatory neuroimaging in the presence of blood-based markers. The subjects were classified as having MCI if they met all of the following criteria at baseline: 1) a CDR score <1; 2) normal daily functioning; and 3) a score greater than or equal to four for the word list delayed recall task on the ADAS-Cog-13 assessment, which coincides with a score <1 SD below the mean for the general age group.^44^ Subjects without either a dementia diagnosis or impaired cognitive performance were labeled as HC for healthy control.

### Cognitive scores and the derivation of cognitive domain-specific z-scores

The ADAS-Cog-13 subscale was originally developed as an extension of the original 11-item ADAS-Cog, which added tasks to assess attention and concentration, planning and executive function, verbal and nonverbal memory, and praxis.^33^ ^,^ ^45^ Scores ranging from 0 to 85, with higher scores indicative of higher cognitive impairment.

To examine whether performance in the two early-impaired AD-specific cognitive domains of memory and executive function is associated with microbiome composition, we created composite z-scores to ease interpretation and comparison across domains. For our memory z-score, we calculated the sum at each sample point of the word recall score, word recognition score, orientation score, remembering word recognition score, and delayed recall score. The mean and standard deviation of the sum score of all the subjects at each visit time were calculated. A z-score for each visit was calculated by subtracting the mean memory sum score of the visit from the memory sum score of a subject and dividing it by the standard deviation of that visit. An executive function z-score was found by first summing the executive function maze time score, maze number of mistake score, dimensional card sort task (NIH toolbox), and the pattern comparison processing speed test score (NIH toolbox). The mean and standard deviation of each group of tests were calculated for each visit group and used to calculate a visit and sample-specific z-score. For both measures, a higher value is indicative of greater cognitive impairment.

### Sample handling and DNA sequencing

Stool samples were collected at home and mailed to the laboratory. All stool samples were collected as part of the participant's normal bowel habits from defecation using the Fisherbrand™ Commode Specimen Collection System and the sample was placed into the OMNIgene•GUT collection kits (DNAgenotek, catalog no. OMR-200), which stabilizes the sample at room temperature for shipment via mail service.

Samples were stored at −80 °C until DNA extraction and sequencing were performed. DNA was extracted from samples using the QIAGEN DNAeasy PowerSoil kits (QIAGEN, catalog no. 47016). Sequencing libraries were constructed using the Nextera XT DNA Library Prep Kit. Libraries were sequenced on a NextSeq 500 or NextSeq 1000 system with 2 × 150 nucleotide paired-end reads.

Quality control was performed on sequencing reads using the KneadData pipeline (https://github.com/biobakery/kneaddata) version v0.12.0, which trims low-quality reads and filters out host contamination sequences (aligned to the human reference version hg37dec_v0.1). Metagenomic reads were profiled for microbial abundance, metabolic pathways, and other functional measures using MetaPhlAn4 and HUMAnN3 databases and tools.^46^ ^,^ ^47^ Sequences were also annotated using the MetaCyc^48^ and KEGG^49^ databases for analysis of functional metabolic pathways.

### Microbiome analysis and modeling

#### Clinical covariate contribution to changing microbiome composition using MERF

As performed in,^50^ mixed-effect random forest (MERF) regression modeling was used to determine the contribution of clinical covariates (see Figure 2) to microbial composition. To minimize output leakage in modeling, we specifically excluded medications and medication classes, as many medications are known to affect the microbiome. For every microbe, the relative abundance was predicted as a function of clinical covariates, and the participant ID as a random effect. Permutated variable importance was used to determine which clinical covariates were significantly informative of microbiome composition (*p*-value cut-off < 0.05).

#### Linear mixed-effect modeling for diversity and cognitive status

To identify the influence of sex, age, education, antibiotic exposure, time since enrollment, and cognitive status on microbiome diversity (inverse Simpson), we implemented a linear mixed-effects model with the following formula: Diversity~Sex+Age+YearsofEducation+AntibioticUsage+Time+CogStatus+1|ID .

Linear mixed-effect models were implemented with the nlme R package (version 3.1.164). The random effect 1|ID was included to account for individual variation. The alpha diversity index, inverse Simpson index, was calculated using the phyloseq R package (version 1.42.0). Sex represents what the subject identifies as at enrollment. In this study, all the participants identified with what they were assigned at birth. Antibiotic usage showed whether the subject had used antibiotics in the 6 months preceding sampling, and the time represented days since enrollment.

To assess the stability of the cognitive scores over time, we again implemented linear mixed-effect models. For the entire GAINS cohort, the following formula was used: Cognitivescore~Time+Cog.Status+1|ID .

#### MERF modeling of ADAS-Cog-13 and cognitive domain z-scores

To find associations between cognitive scores and microbiome features, we developed an MERF regression pipeline. The first step of this pipeline was to split the longitudinal data into a training and a test set by randomly leaving out one sample from every subject from the training set to be used to build the test set. To prevent possible outcome leakage, minimal clinical covariates were used, which included the following: sex, age, antibiotic exposure during the preceding 6 months, hospitalizations during the preceding 6 months, malnutrition score, clinical frailty score, polypharmacy, and years of education. Feature selection was first performed on the training data using the Boruta algorithm^51^ (package version 9.0.0). The selected features were then used to build an MERF model.^52^ The performance of the models is presented as actual versus predicted graphs, where the predicted data are from the test dataset for each seed. The procedure was repeated ten times, corresponding to ten different random seeds. A final version of the model was generated using all Boruta-selected features across the ten seeds and all subject samples. Permutated variable importance of the features contributing to the predictions was then run using the final model (R package vita, version 1.0.0)^53^ to determine a qualitative ranking of the features in the reduced feature space. To analyze the relationships between individual features and scores, Spearman correlation was calculated for each feature. Multiple correction testing on correlations was performed by calculating the false discovery rate (FDR), which matched well with *p*-values for correlations. All analyses and visualizations were performed using R (version 4.2.1).

## Results

### Characteristics of the GAINS cohort participants

Participants were recruited to the GAINS from community centers and physicians' offices across central Massachusetts. Based on information collected at the first clinic visit, participants were categorized into one of three cognitive status groups: cognitively normal controls (HC, *n* = 158), those with mild cognitive impairment (MCI, *n* = 40), and those with Alzheimer's disease (AD, *n* = 25) (see Methods section for classification details). Over 4 y, these 223 participants provided fecal samples at least once, with an average of 3.8 (SD 2.4) samples collected per participant for a total of 856 samples. The average age of the participants was 71.5 y (SD 7.5), but age differed significantly between the cognitive groups, with MCI being older and HC being younger on average (Table 1). A total of 63.7% of the total GAINS cohort was female, with more females in the HC group and roughly equivalent sex ratios in the MCI and AD groups (Table 1).

**Table 1.** GAINS patient demographics and clinical scores.

Elder Characteristic^a^	HC	MCI	AD	*p*-value
	(*n* = 158)	(*n* = 40)	(*n* = 25)	
Age (mean [SD]) (y)	70.2 (7.46)	75.1 (7.22)	73.9 (5.75)	<0.001
**Sex**				
Female	109 (69.0%)	21 (52.5%)	12 (48.0%)	0.0805
Male	49 (31.0%)	19 (47.5%)	13 (52.0%)	
**Polypharmacy**				
Less than five medications	115 (72.8%)	24 (60.0%)	8 (32.0%)	<0.001
Five or more medications	43 (27.2%)	16 (40.0%)	17 (68.0%)	
Antibiotics (past 6 months)	28 (17.7%)	9 (22.5%)	2 (8.0%)	0.475
Hospital exposure	13 (8.2%)	4 (10.0%)	1 (4.0%)	0.753
**Clinical Scores**				
CFS (mean [SD])	2.07 (1.02)	2.54 (0.942)	3.52 (1.53)	<0.001
MIS (mean [SD])	1.21 (0.470)	1.28 (0.510)	1.68 (0.627)	<0.001
ADAS-Cog-13 (mean [SD])	9.01 (4.42)	21.0 (12.8)	32.3 (20.1)	<0.001

CFS = clinical frailty score; MIS = malnutrition index score.

^a^ Data are presented as the number (%), unless otherwise specified.

Polypharmacy, known to be associated with dementia^54^ and defined as taking five or more regularly prescribed medications, was prevalent in 34.1% of participants, with the highest percentage (68%) in the AD group and lowest percentage (27.2%) among HCs. The incidence of polypharmacy was statistically significant between the cognitive groups. A total of 17.5% of all participants were exposed to antibiotics in the 6 months preceding sampling (Table 1), with the MCI group having the highest exposure (22.5%). The prevalence of all daily medications is listed in Supplemental Table 1.

**Figure 1.**
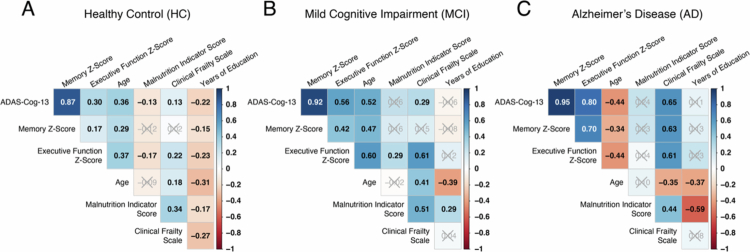
Spearman correlation coefficients between each cognitive outcome (ADAS-Cog-13, Memory Z-score, and Executive Function Z-scores) and relevant clinical covariates in (A) cognitively normal healthy controls, (B) mildly cognitively impaired, and (C) AD diagnosed participants.

Worse clinical frailty and malnutrition indicator scores correlated significantly with cognitive status (Table 1). Both frailty and malnutrition are related to AD and mild cognitive impairment.^55-58^ Clinical frailty was also positively correlated with age and malnutrition in the HC and MCI groups (Figure 1). Among participants with AD, clinical frailty was positively correlated with malnutrition but negatively correlated with age, indicating that younger patients in this group were frailer. Age did not correlate significantly with malnutrition in any of the three cognitive groups (Figure 1).

### Cognitive measures correlated with demographics and clinical covariates and showed stability in AD participants

A battery of cognitive testing was performed at each visit, providing us with three measures of interest: ADAS-Cog-13 score, memory z-score, and executive function (EF) z-score. The ADAS-Cog-13 is a global measure of cognitive function with a specific emphasis on domains implicated in AD progression,^59^ including memory, language, executive function, attention, and praxis. As expected, there were statistically significant differences in ADAS-Cog-13 scores, with the AD group scoring highest (worse cognition) and HC group scoring lowest (Table 1). As memory and executive function represent domains profoundly affected in AD during the early clinical stages, we focused on these two domains by calculating composite z-scores for memory and executive function using components of the ADAS-Cog-13 test and the NIH toolbox (see Methods for further explanation of derivation). Like the ADAS-Cog-13 score, higher memory and EF z-score values indicate greater impairment in the given domain or worse performance.

Performance on cognitive tests can be influenced by a myriad of factors, including age and years of education.^60^ In the HC and MCI groups, age was correlated with higher scores for all three cognitive measures (Figure 1A, B). In participants with AD, age was correlated with lower scores for all tests, suggesting that younger participants are more severely impaired (Figure 1C). Our MCI group will naturally consist of individuals who are experiencing “normal” aging as well as individuals who will progress to AD and other forms of dementia, which we cannot distinguish in the current study. Considering that our MCI group is overall older than the HC and AD groups, our observation of a matching direction of correlation between age and cognitive performance between the HC and MCI groups and an inverse correlation between the MCI and AD groups may indicate that our MCI group consists of a higher proportion of “normal agers.” A negative correlation was found between years of education and cognitive score in the HC cohort but not in the MCI or AD groups (Figure 1). Frailty was positively correlated with all scores in the AD group; in the MCI and HC groups, frailty was positively correlated with ADAS-Cog-13 and EF z-scores (Figure 1). Malnutrition was negatively correlated with ADAS-Cog-13 and EF z-scores in the HC cohort (Figure 1A). Altogether, our analysis suggests that among HC participants, health and education measures are more informative of cognitive scores than are participants with AD. In the AD group, younger age and increased frailty correlated with cognitive scores.

### Microbial composition is informed by demographics and clinical factors in the GAINS cohort

**Figure 2.**
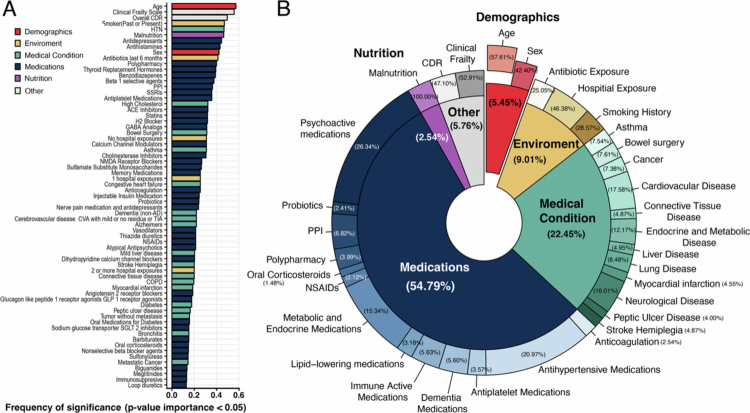
Clinical and demographic variables that predict microbiome composition in community-dwelling individuals. MERF modeling results using all demographics and clinical variables (including each medication taken, presence of polypharmacy, comorbidities, and clinical scoring of frailty and nutrition) to predict each species abundance from repeated fecal samples. (A) Frequency-based ranking of predictors from MERF models. Significance was determined by running permutated variable importance analysis and using an FDR-adjusted *p*-value of 0.05. (B) Pie chart illustrating the distribution of the clinical factors found by the model to be significantly associated with the GAINS microbiome by taxonomy. Drugs were grouped by treatment class.

Within the GAINS cohort, alpha diversity, a general metric of microbiome composition within individual samples, was not significantly different across different cognitive statuses (Supplemental Figure 1). However, antibiotic usage within the past 6 months prior to sampling was significantly associated with lower diversity (Supplemental Table 2). To examine associations between microbial abundance and clinical covariates in the GAINS cohort, we ran mixed-effect random forest (MERF) modeling to predict each species abundance as a function of demographics and clinical covariates (Figure 2). A mix of demographic, medication, and environmental factors was significantly predictive of microbial abundance across the 311 microbe-specific models (FDR-adjusted *p*-value < 0.05; Figure 2). While age was the most frequently selected feature (appearing in 57.2% of models), medication accounted for 54.79% of the significant predictors. Specifically, psychoactive and antihypertensive medications were most prevalently associated with microbiome abundance.

### Depletion of some health-associated microbes is informative of worse ADAS-Cog-13 scores in individuals with MCI and AD

**Figure 3.**
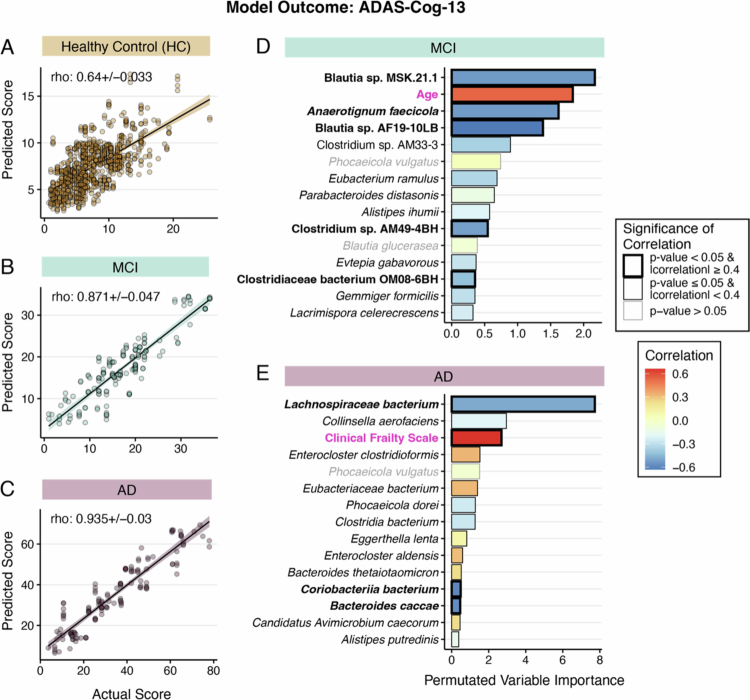
Mixed-effect random forest (MERF) regression models using species abundance and clinical covariates to predict ADAS-Cog-13 scores. Actual versus predicted ADAS-Cog-13 values for (A) cognitively normal healthy control, (B) MCI, and (C) AD patient populations demonstrate good correlation between the predicted and actual scores in MCI and AD groups. Healthy controls were not examined further because of the low association between the predicted and actual values. Permutated variable importance for the final model for the (D) MCI and (E) AD populations. The bar color (D and E) corresponds to the correlation coefficient of each variable with ADAS-Cog-13 score, with red indicating a positive correlation and blue indicating a negative correlation. Magenta font highlights the clinical covariates. The significance of the correlation is indicated by gray (where *p*-value > 0.05) or a black outline (*p*-value ≤ 0.05; FDR equivalent < 0.1). For *p* ≤ 0.05, the magnitude of correlation is indicated by the weight of the outline (heavier outline for |correlation| ≥ 0.4).

We next sought to identify microbiome features associated with cognitive scores between the patient groups. As above, we built MERF regression models for HC, MCI, and AD patients separately, beginning with species abundance combined with demographics and clinical covariates. Models predicting ADAS-Cog-13 scores in the HC group produced marginally informative results, as evident by the correlation between actual and predicted ADAS-Cog-13 for the species abundance-based model, 0.64 ± 0.05 (Figure 3A, Supplemental Table 3). Conversely, models trained on MCI and AD subsets demonstrated strong predictive performance, with correlation coefficients of 0.87 ± 0.05 and 0.94 ± 0.03, respectively (Figure 3B, C and Supplemental Table 3). These results may reflect the functional design of the ADAS-Cog-13, which is powered to identify differences in MCI and mild AD cases and thus focuses on AD-specific cognitive domains. The test is thus not developed or powered to assess general cognition in healthy individuals.

As the MCI and AD patient's subsets cognitive performance-microbiome model displayed strong predictive power, we sought to determine the most informative microbial species of the predicted signal by analyzing the permutated importance ranking of features used in the model. We found that the leading microbial predictors of ADAS-Cog-13 scores in the MCI group included two *Blautia* representatives, a known health-associated Clostridium Cluster IV/XIVa SCFA-producing bacterium,^61^ (ranked 1st and 4th) and *Anaerotignum faecicola* (ranked 3rd), all of which showed significantly (*p*-value < 0.05) lower abundance that strongly correlated (|correlation| ≥ 0.4) with worse(higher) ADAS-Cog-13 scores (Figure 3D and Supplemental Figure 2A). Among participants with MCI, the only clinical covariate in the top fifteen predictors correlated with higher ADAS-Cog-13 scores was increased age (ranked 2nd), suggesting that general age-related cognitive decline may be occurring in the MCI group.

A lower abundance of *Lachnospiraceae bacterium* (also a Clostridium cluster XIVa member)*, Coriobacteriia bacterium,* and *Bacteroides caccae* were found to be the top predictors of worse ADAS-Cog-13 scores in the AD group (Figure 3E and Supplemental Figure 3A). Corroborating the finding that frailty strongly correlates with ADAS-Cog-13 scores (as in Figure 2C), the model identified higher clinical frailty as a stronger predictor of higher ADAS-Cog-13 scores in AD patients (ranked 3rd, and only the clinical variable in the top fifteen predictors).

### Microbiome species associated with performance in cognitive subdomains, though with unclear functional implications

**Figure 4.**
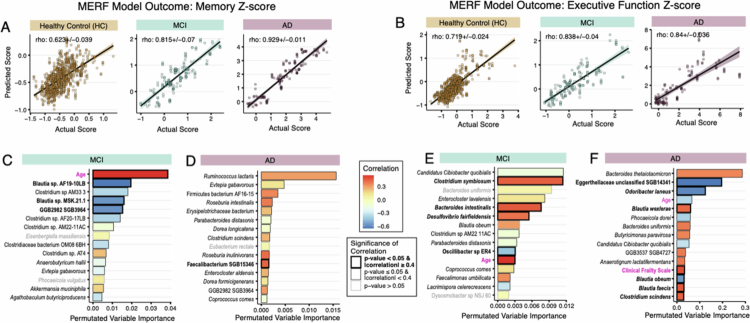
MERF regression models using species abundance and clinical covariates to predict memory and executive function z-scores. Actual versus predicted memory z-score values for cognitively normal healthy control (HC), mild cognitive impairment (MCI), and Alzheimer's disease (AD) patient populations for (A) memory Z-score and (B) executive function Z-score. Permutated variable importance for the final models for the MCI and AD populations for (C and D) memory Z-score and (E and F) executive function Z-score. Magenta font highlights the clinical covariates. The bar color (C–F) corresponds to the correlation coefficient of each variable, where red indicates a positive correlation and blue indicates a negative correlation. The significance of the correlation is indicated by gray (where *p*-value > 0.05) or a black outline (*p*-value ≤ 0.05; FDR equivalent < 0.1). For *p* ≤ 0.05, the magnitude of correlation is indicated by the weight of the outline (heavier outline for |correlation| ≥ 0.4).

We next sought to determine whether we could disentangle the predictive ability of microbial features seen for ADAS-Cog-13 to memory and EF specifically, since these cognitive domains are affected earlier in clinical disease. MERF models trained to predict memory z-scores from species abundance in combination with demographics and clinical features predicted scores that tightly correlated with the actual scores for both the MCI (correlation = 0.815 ± 0.07) and AD (correlation = 0.929 ± 0.01) populations (Figure 4A). The predictions of EF z-scores were also well correlated with the actual scores in both the MCI (0.838 ± 0.04) and AD populations (0.84 ± 0.04) (Figure 4B). Predictions in the cognitively normal HC population were again subpar and thus not further examined (Figure 4A, B).

We again evaluated features that contributed most to the MERF predictions by permutated importance analysis. As with the MERF models for ADAS-Cog-13 scores, nearly all the top predictors were microbial features. In the MERF model for predicting memory z-scores for the MCI group, two *Blautia* species and an uncultured microbe (GGB2982 SGB3964) were identified as the top predictors with strong negative correlation (*p*-value < 0.05 and |correlation| ≥ 0.4) (Figure 4C and Supplemental Figure 2B). In the MERF model predicting memory z-scores for the AD group, only one microbial representative (*Faecalibacterium* SGB15346) was identified as predictive and significant with strong correlation, ranking 11th. All top predicting microbes for memory z-scores in the AD group correlated positively (Figure 4D and Supplemental Figure 3B). Only two of the fifteen top predictors for memory z-scores were common between the MCI and AD groups (GGB2982 SGB3964 and *Evtepia gabavorous*) but showed opposite correlations (Figure 4C, D).

In the MERF model predicting EF z-score for individuals with MCI, higher abundance of three bacterial species (*Clostridium symbiosum, Bacteroides intestinalis,* and *Desulfovibrio fairfieldensis*) associated with inflammation^62-64^ was identified as significant with strong positive correlations. A lower abundance of one health-associated microbe (*Oscillibacter* sp. ER4)^65^ was identified as significantly and strongly correlated (negative correlation) (Figure 4E and Supplemental Figure 2C). In the model predicting EF z-score for the AD group, six bacteria were significant top predictors with strong correlations (Figure 4F and Supplemental Figure 3C). A lower abundance of an Egerthellaceae species, *Odoribacter laneus*, and *Blautia obeum* were correlated with worse EF z-scores (negatively correlated), while higher abundances of *Blautia wexlerae, Blautia faecis,* and *Clostridium scindens* were predictive of worse EF scores (positively correlated). Between the MCI and AD groups, only three microbial features were common among the top fifteen predictors for EF z-score (*Candidatus Cibiobacter qucibialis*, *Bacteroides uniformis*, and *B. obeum*), two of which showed opposite correlations between the MCI and AD groups (Figure 4E, F). These species have been associated with both pro- and anti-inflammatory activities, making their functional correlations here unclear.^66-69^

Only two demographic/clinical features were identified among the top fifteen predictors in these models. Age was selected as a top predictor of both the memory z-score and EF z-score in the MCI population (Figure 4C, E), which matched our analysis of clinical covariate correlations (Figure 1B). Like the ADAS-Cog-13 score, increased age was significantly correlated with worse memory and EF z-scores, suggesting that some impairment may result from age-related cognitive impairment in the MCI population. To this end, the MCI group was significantly older (75.1 ± 7.22 y; *p*-value < 0.001) than both the HC (70.2 ± 7.46 y) and AD groups (73.9 ± 5.75 y). In the MERF model predicting EF z-scores among participants with AD, both age and clinical frailty were identified as among the top fifteen predictors. Age was negatively correlated, though not significant, while clinical frailty was positively correlated and significantly correlated (*p*-value < 0.05 and |correlation| ≥ 0.4) (Figure 4F). These correlates again matched our earlier analyses (Figure 1C) and suggest that, unlike the MCI group, younger participants with AD seem to have worse cognition.

**Figure 5.**
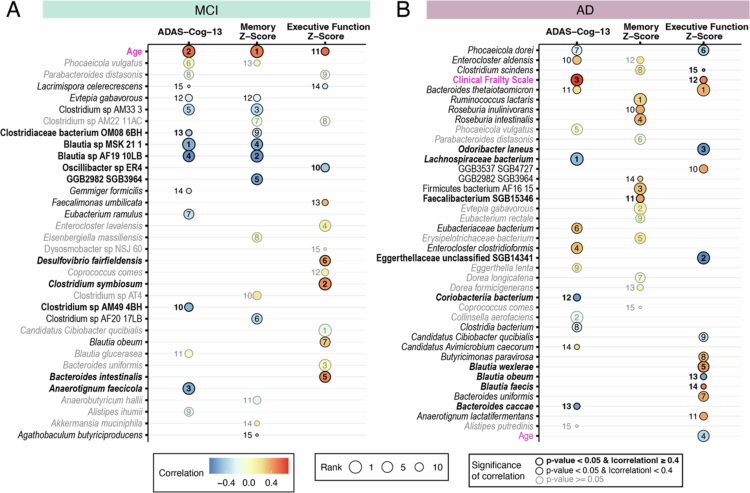
Comparison of rank and correlation of top 15 permutated variables from species abundance-based MERF models for each cognitive outcome. Models trained with (A) the MCI and (B) AD subsets. The circle size and number indicate the rank. The color within the circles corresponds to the correlation coefficient of each variable, where red indicates a positive correlation and blue indicates a negative correlation. Magenta font highlights the clinical covariates. The gray font and circle outlines indicate non-significant correlation (*p*-value ≥ 0.05). Significant *p*-values for correlations are indicated in black font and outlines (*p*-value ≤ 0.05; FDR equivalent < 0.1). The magnitude of correlations is indicated by the weight of the outline (heavier outline for |correlation| ≥ 0.4).

Examining the top-ranked important features across the MERF models for the three cognitive measures, only a few predictors ranked consistently as predictive and significantly correlated (Figure 5). Among participants with MCI, age was consistently selected as a predictor with a strong and significant correlation (Figure 5A). Two *Blautia* members (*Blautia* sp. MSK 21 and *Blautia* sp. AF19 10LB) were identified as predictors with significant correlations for both ADAS-Cog-13 and memory z-scores. For EF z-scores, none of the species identified as predictive with significant correlation were selected as the top predictors for ADAS-Cog-13 or memory z-scores. Among participants with AD, no microbial or clinical features ranked as a top predictor in all three cognitive measures. Only four species ranked in two of the three tests. We also failed to find consistent patterns for pro- versus anti-inflammatory-associated microbes across the three cognitive measures for the MCI and AD groups. Altogether, these results lead us to hypothesize that it is not the individual species present, but rather the functions of the microbiome that are shared across different microbes or composite microbial communities predict cognitive functioning.

### Enrichment of pathways involved in the metabolism of arginine, glutamate, lysine, and sialic acid is informative of cognitive performance in participants with MCI

While the results are informative, microbial abundance alone fails to provide insight into the functionality of the microbial community. Analyses of microbial metabolic pathway genes enable a more complete view of the functional microbiome or the function of genes found in fecal samples that produce metabolites in the gut and interact with or influence the host. Analysis of metabolic pathways may also help reconcile incongruous findings in species abundances (i.e., different species or communities with similar functional gene pathways may produce the same metabolites). Accordingly, we profiled the abundances of microbial-encoded metabolic pathways (using the MetaCyc database) from our sequenced microbiome samples. Metabolic gene pathway abundances were determined by aligning our sequencing results against the MetaCyc database.

**Figure 6.**
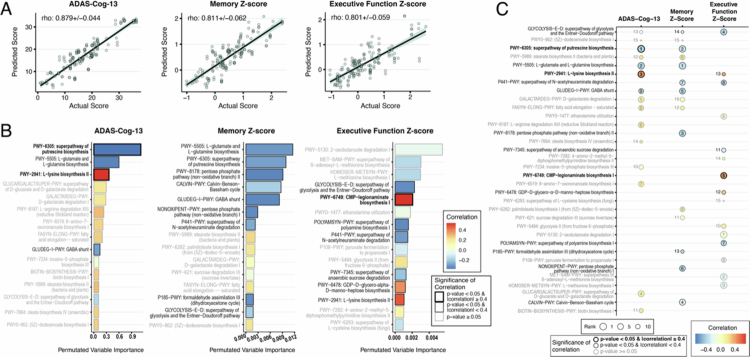
Pathway abundance analyses using MetaCyc in MCI population. Comparison of the rank and correlation of the top 15 permutated variables from pathway abundance-based MERF models for each cognitive outcome. Models trained with the MCI subset to (A) predict ADAS-Cog-13, memory z-score, and executive function z-score. Top 15 predictors (B) from ADAS-Cog-13, memory z scores, and executive function z scores colored for correlation coefficient of each pathway with the identified outcome. The bar color corresponds to the correlation coefficient of each variable, where red indicates a positive correlation and blue indicates a negative correlation. The significance of the correlation is indicated by gray (where *p*-value > 0.05) or a black outline (*p*-value ≤ 0.05; FDR equivalent < 0.1). For *p* ≤ 0.05, the magnitude of correlation is indicated by the weight of the outline (heavier outline for |correlation| ≥ 0.4). (C) Comparison of the rank and correlation of the top 15 permutated variables from pathway abundance-based MERF models in each cognitive outcome for the AD population. The color within the circles corresponds to the correlation coefficient of each variable, where red indicates a positive correlation and blue indicates a negative correlation. The gray font and circle outlines indicate non-significant correlation (*p*-value ≥ 0.05). Significant *p*-values for correlations are indicated in black font and outlines (*p*-value ≤ 0.05; FDR equivalent < 0.1). Magnitude of correlations indicated by the weight of the outline (heavier outline for |correlation| ≥ 0.4).

Starting with the MCI group, we again utilized MERF models to predict ADAS-Cog-13, memory z-scores, and EF z-scores using a combination of basic clinical covariates and metabolic pathway relative abundance, and analyzed pathways contributing to the predictions by permutated importance. The predicted values correlated well with the actual cognitive scores, similar to our microbial abundance analysis above (ADAS-Cog-13 correlation = 0.879 ± 0.044; memory z-score correlation = 0.811 ± 0.062; EF z-score correlation = 0.801 ± 0.059) (Figure 6A). In the model predicting ADAS-Cog-13 scores, four pathways were selected among the top fifteen predictors, with two strongly correlated pathways (Figure 6B, left panel and Supplemental Figure 4A). Putrescine biosynthesis (PWY-6305) showed a strong and significant negative correlation, while L-lysine biosynthesis (PWY-2941) showed a strong and significant positive correlation. The putrescine biosynthesis pathway involves the metabolism of arginine and its metabolite ornithine as well as the production of urea, which can be a nitrogen source for gut bacteria.^70^ ^,^ ^71^ The other two significant pathways (PWY-5505 and GLUDEG-I-PWY) are both related to the metabolism of glutamate, which can act as a potent neurotransmitter and be converted to the neurotransmitter GABA in mammalian cells.

In the MERF model predicting memory z-scores, lower abundance of several pathways was selected as top predictors, though none with strong correlations (Figure 6B, middle panel and Supplemental Figure 4B). These include the same putrescine biosynthesis and glutamate pathways selected in the ADAS-Cog-13 model as well as pathways involved in glycolysis (including pentose phosphate pathways), N-acetylneuraminate metabolism, and formaldehyde assimilation, indicating potential carbon sources for gut bacteria.

In the MERF model predicting EF z-scores, only one pathway was identified as significant with a strong correlation, a CMP-legionaminate biosynthesis pathway (PWY-6749) (Figure 6B, right panel and Supplemental Figure 4C). This pathway involves glutamate as intermediaries to produce sialic acid derivatives, which are used by some pathogens in virulence and host immune evasion. Higher abundance is correlated with worse EF z-scores, possibly indicating a contribution by potential pathobionts. Pathways involved in lysine and heptose biosynthesis were also significantly positively correlated, though not with a strong correlation. Heptose sugars are important components of LPS in most gram-negative bacteria. A lower abundance of pathways in glycolysis, polyamine biosynthesis, N-acetylneuraminate metabolism, and anaerobic sucrose degradation was also significantly correlated.

Across the three cognitive measures, a lower abundance of a glycolysis pathway was selected as common among the top 15 predictors, although it was not strongly correlated (Figure 6C). Nine pathways were common between the two cognitive tests, with correlations in the same direction. Significant pathways include putrescine biosynthesis, glutamate metabolism, lysine biosynthesis, and N-acetylneuraminate degradation.

### Enrichment of nitrogen-metabolizing and methionine-related pathways is informative of cognitive performance in participants with AD

**Figure 7.**
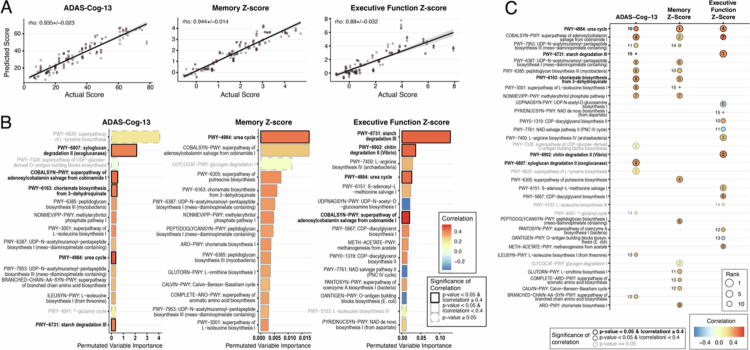
Pathway abundance analyses using MetaCyc in AD population. Comparison of the rank and correlation of top 15 permutated variables from pathway abundance-based MERF models for each cognitive outcome. Models trained with the AD subset to (A) predict ADAS-Cog-13, memory z-score, and executive function z-score. Top 15 predictors (B) from ADAS-Cog-13, memory z-score, and executive function z-score colored for correlation coefficient of each pathway with the identified outcome. The bar color corresponds to the correlation coefficient of each variable, where red indicates a positive correlation and blue indicates a negative correlation. The significance of the correlation is indicated by gray (where *p*-value > 0.05) or a black outline (*p*-value ≤ 0.05). For *p* ≤ 0.05, the magnitude of correlation is indicated by the weight of the outline (heavier outline for |correlation| ≥ 0.4). (C) Comparison of the rank and correlation of top 15 permutated variables from pathway abundance-based MERF models in each cognitive outcome for the AD population. The color within the circles corresponds to the correlation coefficient of each variable, where red indicates a positive correlation and blue indicates a negative correlation. The gray font and circle outlines indicate non-significant correlation (*p*-value ≥ 0.05). Significant *p*-values for correlations are indicated in black font and outlines (*p*-value ≤ 0.05; FDR equivalent < 0.1). The magnitude of correlations is indicated by the weight of the outline (heavier outline for |correlation| ≥ 0.4).

For the AD group, MERF models predicting ADAS-Cog-13, memory z-scores, and EF z-scores also predicted values that were tightly correlated with actual scores (ADAS-Cog-13 correlation = 0.935 ± 0.02; Memory z-score correlation = 0.944 ± 0.01; EF z-score correlation = 0.88 ± 0.03) (Figure 7A).

In the model predicting ADAS-Cog-13 scores, all fifteen top predictors showed positive correlations. Higher abundances of five pathways were identified as significant with strong correlations (Figure 7B, left panel and Supplemental Figure 5A), including pathways involved in xyloglucan degradation (PWY-6807), vitamin B12 cofactor salvaging (COBALSYN-PWY), chorsimate biosynthesis (PWY-6163), the urea cycle (PWY-4984), and starch degradation (PWY-6731). In the model predicting memory z-scores, all of the top fifteen predictors also showed positive correlations; only one pathway, the urea cycle, was significant (Figure 7B, middle panel and Supplemental Figure 5B). In the model predicting EF z-scores, most of the top fifteen predictors showed positive correlations. Higher abundance of four pathways was identified as significant with strong correlations (Figure 7B, right panel and Supplemental Figure 5C), including starch (PWY-6731) and chitin (PWY-6902) degradation.

Two pathways were selected as important across all three cognitive outcome models. Notably, the urea cycle (PWY-4984) was strongly and significantly correlated with worse performance in each cognitive outcome (Figure 7C), ranking 10th for ADAS-Cog-13, 1st for the memory z-score, and 4th for EF z-score. Few species in our AD samples confidently contributed to the abundance of the urea pathway (Supplemental Figure 8A). Urea is also a significant source of nitrogen for gut bacteria.^72^ ^,^ ^73^ The pathway for vitamin B12 cofactor salvaging (COBALSYN-PWY) was also selected as important for all three outcomes, with higher abundance correlated with worse scores, with strong correlations were strong for ADAS-Cog-13 and EF z-scores. The vitamin B12 cofactor (adenosylcobalamin), the active form of vitamin B12 in the body, is a cofactor for highly conserved enzymes involved in amino acid synthesis and the breakdown of fatty and amino acids (Rowley and Kendall, 2019). A combination of commensals and few pathobionants contributed to the abundance of the vitamin B12 cofactor salvage pathway (Supplemental Figure 8B). Gut bacteria also use B12 as a critical cofactor for many metabolic reactions, including the production of methionine.^74^

Several pathways were selected as significantly important features contributing to both worse (higher) ADAS-Cog-13 and memory z-scores in MERF models (positive correlations), though none met the strong correlation cutoff of 0.4. These include pathways involved in the biosynthesis of peptidoglycan (PWY-7953, PWY-6387, and PWY-6385), chorismate (PWY-6163), isoleucine (PWY-3001), and isopentenyl pyrophosphate (NONMEVIPP-PWY). Peptidoglycan is an important and major component of bacterial cell walls. Chorismate is an intermediary for the synthesis of aromatic amino acids (i.e., phenylalanine, tyrosine, and tryptophan). Isopentenyl pyrophosphate is a precursor for the synthesis of isoprenoids, a broad class of compounds essential for both eukaryotes and prokaryotes. Some gut pathogens have been reported to use isoprenoids in virulence and host immune evasion.^75^ ^,^ ^76^ Only one pathway, a pathway for starch degradation (PWY-6731), was common among the top predictors for both ADAS-Cog-13 (ranked 15th) and EF z-score (ranked 1st).

### Microbial genes involved in folate and methionine metabolism are informative of cognitive outcomes

The MetaCyc pathways, as used above, are well curated and highly interpretable. However, understanding the interconnections between pathways and their relationships with specific outcomes often requires complex, manual curation. Thus, to supplement our analyses, we also used the Kyoto Encyclopedia of Genes and Genomes (KEGG) ontology database, which offers broader coverage of pathways and functions that can enable a more holistic view of the microbial functional activity.^77^ KEGG ontology (KO) terms represent functional orthologs that can be linked to a variety of larger pathways and groups. To identify specific gene functions associated with cognitive outcomes in our cohort, we predicted our three cognitive measures using MERF models trained with KO terms encoded by the microbiome in combination with clinical covariates for the MCI and AD groups.

**Figure 8.**
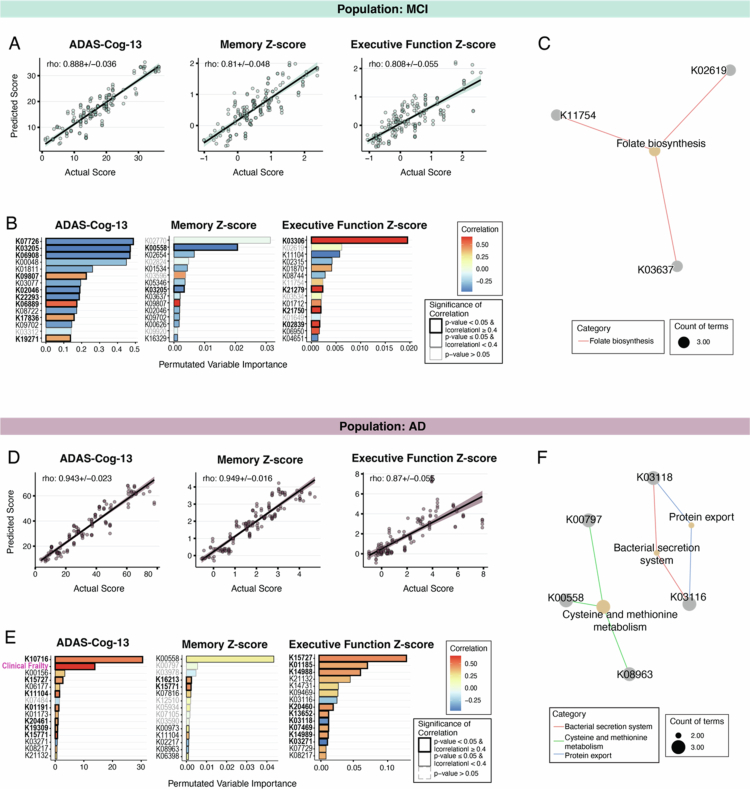
MERF regression models using KEGG Ontology (KO) term abundance and clinical covariates to predict cognitive outcomes in MCI and AD patient populations. (A) Actual versus predicted in AD population for models predicting ADAS-Cog-13, memory z-score, and executive function z-score in participants with MCI. (B) Permutated variable importance for the final model for the ADAS-Cog-13, memory z-score, and executive function z-score. (C) KO term enrichment analysis using top 15 predictors for all three cognitive measures in participants with MCI. (D) Actual versus predicted performance in AD population for models predicting ADAS-Cog-13, memory z-score, and executive function z-score in participants with MCI. (E) Permutated variable importance for the final model for the ADAS-Cog-13, memory z-score, and executive function z-score. (F) KO term enrichment analysis using top 15 predictors for all three cognitive measures in participants with AD. The bar color (B and E) corresponds to the correlation coefficient of each variable, where red indicates a positive correlation and blue indicates a negative correlation. The significance of the correlations (B and E) is indicated by gray (where *p*-value > 0.05) or a black outline (*p*-value ≤ 0.05; FDR equivalent < 0.1). For *p* ≤ 0.05, the magnitude of correlation is indicated by the weight of the outline (heavier outline for |correlation| ≥ 0.4).

For the MCI group, the predicted scores for our three cognitive measures matched well with the actual scores (Figure 8A) (ADAS-Cog-13 correlation = 0.888 ± 0.036, memory z-score correlation = 0.81 ± 0.048, EF z-score correlation = 0.808 ± 0.055). The permeated variable importance for KO terms was calculated as above. Of the top fifteen predictive terms, notable individual KO terms that significantly and strongly correlated (*p* ≤ 0.05 and |correlation| ≥ 0.04) with the outcomes of the cognitive tests included virulence factors and transport proteins (Figure 8B and Supplemental Figure 6). For participants with AD, the actual and predicted ADAS-Cog-13 (correlation = 0.943 ± 0.02), memory z-score (correlation = 0.949 ± 0.02), and EF z-score (correlation = 0.87 ± 0.06) were strongly correlated, indicating high performance by the resulting models (Figure 8D). Notably, the top KO terms with significant and strong correlations in this group included the clinical frailty score and various transport proteins (Figure 8E and Supplemental Figure 7).

To discern patterns in overall functional activity, the top fifteen predictors of each cognitive outcome were combined, and enrichment analysis of the functionality of the selected KO terms was performed for both the MCI and AD groups separately. Genes related to folate biosynthesis were enriched (Figure 8C) in participants with MCI. Among participants with AD, genes related to cysteine and methionine metabolism were significantly enriched, along with genes related to the bacterial secretion system and protein export genes (Figure 8F). Folate biosynthesis and metabolism of cysteine and methionine are connected metabolic pathways that share intermediaries and substrates with vitamin B12.^74^ ^,^ ^78^ The convergence of these pathways in our analyses could indicate a functional role for methionine-related microbial metabolites and/or B vitamins, specifically folate and B12, in the pathogenesis of AD.

## Discussion

In AD, memory loss and executive function deficits are among the important early signs and symptoms of AD.^38^ Here, we examined these subdomains in more depth in the GAINS cohort in an attempt to better understand the contributions of microbial dysbiosis to the cognitive function or dysfunction typical of AD. To create a more comprehensive measure of both memory and executive function, we combined measures within the ADAS-Cog-13 and the NIH toolbox related to these cognitive domains to create a memory z-score and an executive function z-score. Additionally, we integrated metagenomic analysis of the gut microbiome with a detailed assessment of clinical data (i.e., medications and medical comorbidities) and relevant clinical factors such as malnutrition and clinical frailty. This study represents one of the more comprehensive and multifaceted microbiome studies currently targeted at patients diagnosed with or at risk for AD.

In our combined multimodal analyses, microbiome features contributed substantially and independently to the prediction of ADAS-Cog-13, memory z-score, and executive function z-score than clinical features, highlighting the microbiome's importance over individual clinical factors. Age and clinical frailty were the only two clinical factors that were selected as being among the top 15 predictors in any of our MERF models. A potential concern with our modeling approach is that using patientID as a random effect in the MERF framework could introduce data leakage if earlier timepoints are used for training and later timepoints for evaluation where variables such as age and clinical frailty may advance in value. However, this modeling strategy is aligned with clinical goals wherein a patient's accumulated history may be leveraged to forecast their future trajectory (personalized prediction). In this context, it is important to distinguish outcomes from general out-of-sample prediction: the performance reported here should not be interpreted as evidence that models generalize to entirely new, previously unobserved patients. Rather, it reflects the model's ability to capture and extend individual patient trajectories over time. Longer-term follow-up in larger cohorts will shed more light on microbial patterns that distinguish between older adults with MCI who progress to AD versus those who do not.

While our analysis of microbial abundances does add to the field's cumulative literature, we did not find clear or striking patterns by taxa. There is increasing recognition that individual species abundance results can be heterogeneous across microbiome studies for a variety of reasons, including methods of sequencing and analysis, as well as population and geographic heterogeneity. However, “functional” microbiome populations may share common beneficial activities or pathologic traits in the setting of dysbiosis. There is a growing focus on the functional potential of the gut microbiome and measures of metabolism over taxonomic signatures of disease.^79^ Thus, investigating functional gene pathways and metabolic signatures may serve not only to unify study results but also to probe potential mechanisms of disease pathogenesis. We employed two complementary approaches to analyze the contributions of functional gene content within the gut microbiome to cognitive symptoms, the MetaCyc pathway and the KEGG Ontology database. Both methods revealed related patterns centered on specific metabolic pathways with overlapping features (pathways) between the three cognitive measures.

Analysis of the MCI group based on MetaCyc pathways revealed notable pathways in glutamate metabolism and polyamine biosynthesis, which showed negative correlations with worse cognitive scores in general. Glutamate is essential for normal brain function, acting on the N-methyl-d-aspartate receptor (NMDA) receptor, and dysfunction can lead to neurodegeneration.^80^ Dysfunctional production of glutamate from the gut microbiome has been described in AD,^81^ ^,^ ^82^ and evidence here points toward worsening cognitive performance, which is linked to NMDA dysfunction,^83^ as being associated with lower glutamate production from the gut microbiome among MCI patients who are at risk for AD. The role of polyamines, on the other hand, is more multifaceted and complex. Polyamines, including putrescine, appear to be protective to the colonic epithelium by increasing the levels of the SCFA butyrate and decreasing the expression of pro-inflammatory cytokines^84^ as well as by regulating local inflammation.^85^ Microbial polyamines produced in the colon are also absorbed into the bloodstream^86^ where they have the potential to impact systemic host physiology. The polyamine biosynthesis pathways involve catabolism of arginine and its intermediary ornithine and agmatine, specifically for putrescine biosynthesis, as well as catabolism of lysine and S-adenosyl-L-methionine, potentially changing the availability of these upstream metabolites for the host. Altered arginine bioavailability has been reported to be relevant in AD for its role in regulating immune system activation. Arginine is a semi-essential amino acid due to its roles in NO metabolism and central nervous and immune system defenses.^87^ ^,^ ^88^ Arginine is a precursor to nitric oxide; endothelially derived nitric oxide (eNOS) is essential for maintaining cerebral vascular blood flow. Low levels of eNOS in the brain have been associated with the major histopathological hallmarks of AD, Aβ plaques and neurofibrillary tangles,^89^ and low serum arginine and agmatine have been demonstrated in AD patients.^90^ A lack of arginine may drive immune dysfunction, and arginine supplementation has even been proposed as a possible protective mechanism against AD-related neuroinflammation.^91^ ^,^ ^92^ Thus, in the context of AD, the role of microbial polyamines presents a complex picture requiring further investigation. Both of these mechanisms, namely, glutamate biosynthesis and the direct and indirect effects of polyamines, describe how the gut microbiome may mechanistically be linked to worse cognitive performance via the brain and immune dysfunction observed in AD.

Analysis of MetaCyc pathways for the AD group was most notable for the increase of genes in the urea cycle, which was positively associated with worse scores for all three cognitive measures with strong correlations. Urea in the gut lumen is derived mostly from the host, including by active transport from colonocytes.^72^ ^,^ ^73^ ^,^ ^93^ Urea is a major source of nitrogen for gut bacteria, many of which express urease to break down urea into ammonia before recycling into other amino acids and nitrogenous compounds. The upregulation of this pathway may reflect an effort to “salvage” nitrogen sources if amino acids entering from the upper GI track are low. This may occur in the case of generalized malnutrition accompanied by lower protein intake, which is common in older adults. However, our analysis did not find a statistically significant association between malnutrition and cognitive measures, although this has been well reported in the AD literature.^55^ ^,^ ^58^ In the context of AD, increased urease activity in the colon may contribute to local inflammation and breakdown of the gut epithelial barrier. Systemically, uremic toxins also have the potential to disrupt the blood‒brain barrier,^94^ and protein-bound uremic toxins and uremic metabolites have been associated with cognitive function.^95^ ^,^ ^96^ The production of urea in mammalian cells involves catabolism of arginine, ornithine, and citrulline as intermediaries,^97^ adding to the complexity of potential global effects linked to host‒microbiome urea metabolism and cycling. Additionally, dysfunction of the urea cycle in the brain, which is important for the synthesis of neuronal nitric oxide and neurotransmitters such as GABA and glutamate, has been associated with the classic histopathological hallmarks of AD.^98^ These facts link back to our findings in the MCI group, where reduced glutamate and polyamines are associated with worse cognitive performance. Our study suggests that the gut microbial metabolism of urea may contribute negatively to cognitive outcomes and mirrors another recent study in a large cohort of Hispanic adults.^99^

Our analyses of microbial functional genes also revealed the relevance of pathways involved in B vitamins, specifically folate and B12, and cysteine/methionine metabolism in both the MCI and AD groups. MetaCyc analysis identified a pathway of vitamin B12 (adenosylcobalamin) cofactor scavenging, potentially reflective of low B12 levels, as a significant top predictor for all three cognitive measures in the AD group while KEGG analysis identified folate biosynthesis and cysteine and methionine metabolism as important in the MCI and AD groups, respectively. Vitamins B12, folate, cysteine, and methionine are all key components in one-carbon metabolism, a universal mechanism by which methyl donors are generated for numerous cellular processes in both prokaryotes and eukaryotes.^100^ Our combined analyses of metabolic pathways using two different methods have therefore revealed a common metabolic theme. Vitamin B12 is necessary for methionine metabolism,^74^ and low B12 has been mechanistically linked to AD brain pathology.^101^ However, microbial utilization of B12 in the colon is not expected to alter systemic B12 for humans, as dietary B12 is mostly absorbed in the early small intestines, and significant absorption of B12 through colonocytes has not been demonstrated.^102^ Thus, our models point toward higher methionine biosynthesis from the gut microbiome (as opposed to low systemic B12 as a result of microbial consumption) associated with worse cognitive performance. Higher systemic methionine is associated with the development of AD,^103^ and higher methionine leads to increased production of inflammatory cytokines in the brain.^104^ In AD transgenic models, the restriction of methionine led to lower Ab accumulation in APP/PS1 mice.^105^ Thus, dietary methionine restriction has been proposed as an anti-aging intervention for cognitive disorders.^106^ However, most models of methionine restriction come from transgenic mice,^107^ with results showing sex-specific differences.^108^ ^,^ ^109^ Given the known sex differences in the microbiome^110^ as well as AD incidence,^111^ these results could be explained by the gut microbiome. The gut microbiome plays a significant role in the synthesis and breakdown of methionine, which is then available for absorption.^112^ This is demonstrated in studies where removing the microbiome directly alters methionine availability for the host.^113^ Instead of dietary intake, the microbiome dysbiotic state producing excessive methionine may be the critical link “microbiota–gut–brain axis” associated with cognitive decline. Altogether, our results reveal metabolic pathways with potential mechanistic links to cognitive performance in MCI and AD via local, systemic, and neuroinflammatory impacts. Future studies targeting these pathways may help solidify our understanding of the precise triggers for AD pathology and cognitive decline.

### Study strengths and limitations

The strengths of our study include our comprehensive approach to (1) including clinical features in analyses in combination with microbiome data, (2) using multiple objective cognitive assessments, and (3) analyzing metagenomic data using complementary techniques. Together, these aspects should increase confidence in the robustness of our findings, despite limitations to the study, which include a modest size and relatively homogenous sample population due to the geographic location of the study and limited information on patient lifestyle factors (e.g., diet, exercise, social and community supports, etc.), which may improve or worsen dementia symptoms or progression. Related to these aspects is the bi-directionality of these lifestyle contextual factors on the microbiome—e.g., frailer individuals without family, community, or professional support may have poorer diets, which in turn impacts the microbiome. This study is also limited given the absence of host metabolomics, blood‒brain barrier integrity measures, and CSF and plasma markers. Additionally, AD participants were classified based on clinical records as opposed to blood-based biomarkers, which we plan to collect in future studies. The lack of uniform clinical imaging has limited our ability to classify participants as AD based on biological definitions. In this study, we did not confirm a biomarker diagnosis of AD. Due to the longitudinal nature of sample collection and processing, batch effects were unavoidable. However, correction resulted in no substantial changes in the correlations (Supplemental Figure 9). Like all black-box machine learning approaches, MERF models cannot disambiguate the relative contribution of correlated predictors. As age correlates with both microbiome and cognitive outcomes in our cohort, we cannot exclude the possibility that the model is learning an age-mediated relationship. Further, a limitation of MERF models is that the use of a random effect variable based on patients inherently will carry over information relevant to the outcome. Together, these issues represent an inherent model attribution limitation and possible data leakage of the framework used. Further, the stability of features and the models' performance were not evaluated here, as standard feature selection parameters were used. Finally, it is known that medications can shape the microbiome composition, so we cannot clearly state that these pathways do not influence our reported findings. We aim to address these challenges in future studies with expanded cohorts as well as by longitudinal follow-up to replicate and validate our findings.

## Conclusions

In summary, we identify associations between gut microbial metabolic functions and cognitive impairment that are consistent with potential mechanistic links to the level of cognitive impairment in older adults with or at risk of AD. AD is a devastating, progressive disease impacting millions worldwide, with few effective therapies to slow progression. While our study cannot establish causality, these patterns warrant further investigation. If microbial mechanistic links can be identified, they could provide a novel target for therapeutics aimed at slowing this disease. Building upon the field's established associations between AD and the microbiome, our work suggests a potential role for gut microbes involved in the metabolism of polyamines, urea, and methionine in the pathogenesis of AD. This work highlights the complexity of the microbiota‒gut‒brain axis and how multiple mechanisms are likely at play, with potential linkages to each other and ultimately affecting immune dysfunction and inflammation. From our findings, we propose that it is much less about taxonomy and more about how the microbial community functions metabolically and how this in turn interacts with the host, playing a role in exacerbating cognitive performance.

## Supplementary Material

SUPPLEMENTAL FIGURES and CAPTIONS.docx SUPPLEMENTAL FIGURES and CAPTIONS.docx

SUPPLEMENTAL TABLES.docx SUPPLEMENTAL TABLES.docx

## Data Availability

Microbiome sequencing and related metadata are available on NCBI (BioProject ID PRJNA1446836).
