# Supplementary material for: Microbiome functional gene pathways are indicative of cognitive performance in older adults at risk for Alzheimer's disease
Source: Gut Microbes. 2026 May 24;18(1):2676162. doi: 10.1080/19490976.2026.2676162 (PMC13203045; doi:10.1080/19490976.2026.2676162)
Supplement: SUPPLEMENTAL FIGURES and CAPTIONS.docx [file KGMI_A_2676162_SM3144.docx]

SUPPLEMENTAL FIGURES and CAPTIONS

**Supplemental Figure 1:** Shannon and Simpson index values for GAINS participants across the three cognitive status sub populations; cognitively normal healthy controls (HC; orange yellow), mild cognitively impaired (MCI; teal), and diagnosed with Alzheimer’s Disease (AD; violet).

**Supplemental Figure 2: Top predictors selected species abundance in the MCI population by the MERF models.** Species abundance plotted for MCI population against (A) ADAS-Cog-13 scores, (B) Memory Z-scores, and (C)Executive functioning Z-score. Scores on x-axes.

**Supplemental Figure 3: Top predictors selected species abundance in the AD population by the MERF models.** Species abundance plotted for AD population against (A) ADAS-Cog-13 scores, (B) Memory Z-scores, and (C)Executive functioning Z-score. Scores on x-axes.

**Supplemental Figure 4: Top predictors selected microbial-encoded metabolic pathways in the MCI population by the MERF models.** Gene pathway abundances plotted for MCI population against (A) ADAS-Cog-13 scores, (B) Memory Z-scores, and (C)Executive functioning Z-score. Scores on x-axes.

**Supplemental Figure 5: Top predictors selected microbial-encoded metabolic pathways in the AD population by the MERF models.** Gene pathway abundances plotted for AD population against (A) ADAS-Cog-13 scores, (B) Memory Z-scores, and (C)Executive functioning Z-score. Scores on x-axes.

**Supplemental Figure 6: Top predictors selected KEGG ontology terms in the MCI population by the MERF models.** Gene pathway abundances plotted for MCI population against (A) ADAS-Cog-13 scores, (B) Memory Z-scores, and (C)Executive functioning Z-score. Scores on x-axes.

**Supplemental Figure 7: Top predictors selected KEGG ontology terms in the AD population by the MERF models.** Gene pathway abundances plotted for AD population against (A) ADAS-Cog-13 scores, (B) Memory Z-scores, and (C)Executive functioning Z-score. Scores on x-axes.

**Supplemental Figure 8: Species identified contributing to each metabolic pathway using HUMAnN3 annotated pathways.** There were a few species in our AD samples that contributed to the (A) urea cycle pathway and a combination of commensals and pathobionants that contributed the abundance of the (B) vitamin B12 cofactor salvaging pathway. Individual species color coded above with undetermined in black.


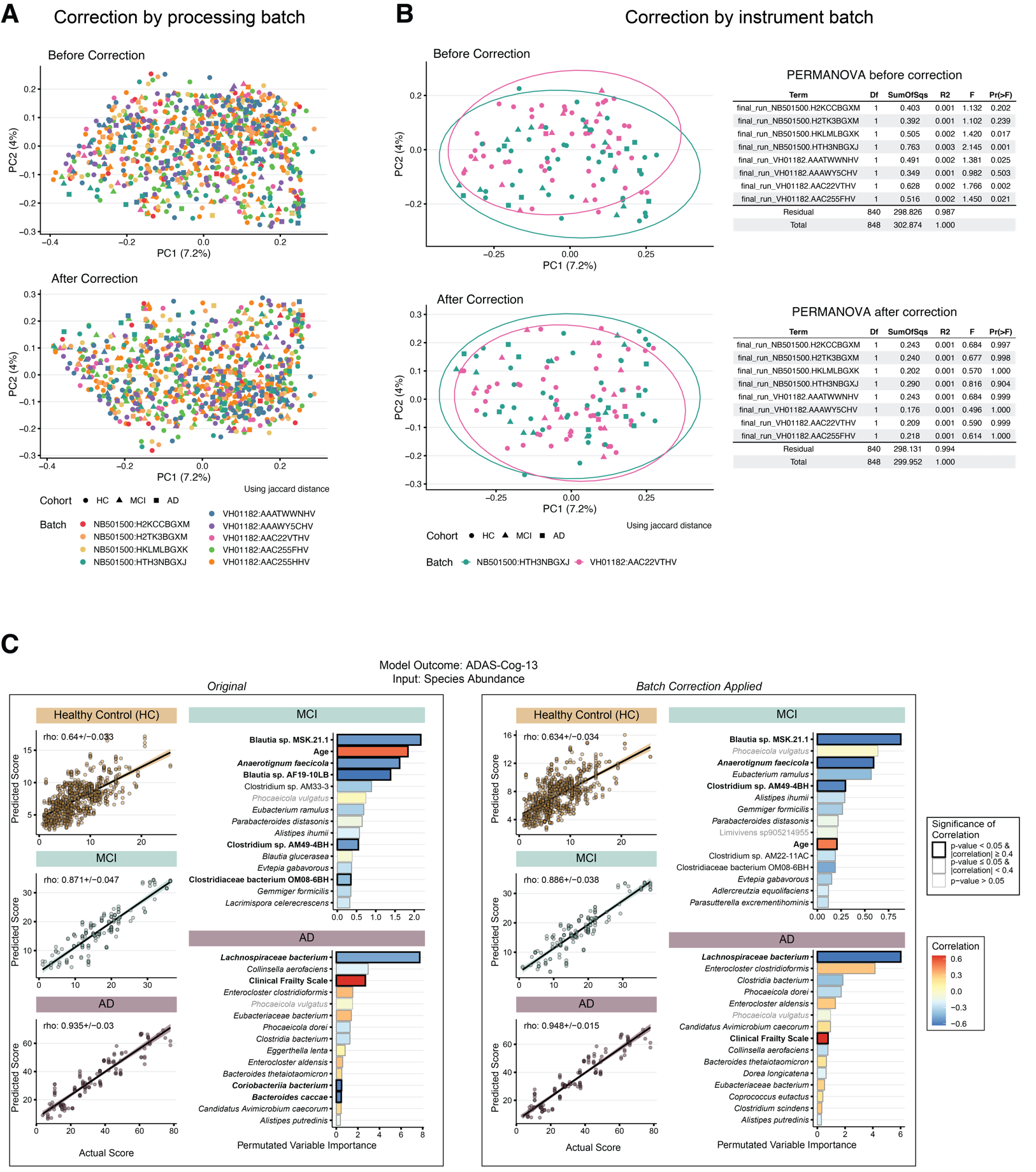


**Supplemental Figure 9: Batch correction applied so species model.** (A) Analysis of sequencing batch effects by processing batch with and without correction using MMUPHin package (<https://huttenhower.sph.harvard.edu/mmuphin/>). (B) Principal Coordinate plots of batches grouped by instrument using Jaccard distances with and without correction using MMUPHin and PERMANOVA comparisons. (C) Comparison of predicted outcomes from ADAS-Cog-13 MERF model trained on species after batch correction using MMUPHin showing no significant changes between predicted versus actual scores, minimal change in correlation strength, and some change rank order for some variables.
