# Supplementary material for: Microbiome functional gene pathways are indicative of cognitive performance in older adults at risk for Alzheimer's disease
Source: Gut Microbes. 2026 May 24;18(1):2676162. doi: 10.1080/19490976.2026.2676162 (PMC13203045; doi:10.1080/19490976.2026.2676162)
Supplement: SUPPLEMENTAL TABLES.docx [file KGMI_A_2676162_SM3143.docx]

SUPPLEMENTAL TABLES

Supplemental Table 1: Daily medications taken by GAINS cohort participants, ordered from most to least prevalent.

| **Class** | **Specific** | **n** | **%** |
| --- | --- | --- | --- |
| **Statins** |  | 104 | 46.6 |
| **Acid reducing medications** | PPI | 53 | 23.8 |
|  | H2 Blocker | 13 | 5.8 |
| **Antidepressants** |  | 38 | 17.0 |
|  | SSRIs | 38 | 17.0 |
|  | Nerve pain medications | 21 | 9.4 |
| **Beta blockers** |  | 34 | 15.2 |
|  | Beta 1 selective agents | 33 | 14.8 |
|  | Nonselective beta blocker agents | 5 | 2.2 |
|  | Beta 1 and 3 selective agonist agents | 2 | 0.9 |
|  | Beta 2 selective agents | 1 | 0.4 |
| **ACE Inhibitors** |  | 34 | 15.2 |
| **Thyroid replacement hormones** |  | 29 | 13.0 |
| **Seizure Medications** |  | 26 | 11.7 |
|  | GABA Analogs | 17 | 7.6 |
|  | Sulfamate Substitute Monosaccharides | 4 | 1.8 |
| **Antiplatelet Medications** |  | 24 | 10.8 |
| **NSAIDs** |  | 20 | 9.0 |
| **Antihistamines** |  | 20 | 9.0 |
| **Calcium channel blockers** |  | 19 | 8.5 |
|  | Dihydropyridine calcium channel blockers | 18 | 8.1 |
|  | Calcium Channel Modulators | 17 | 7.6 |
|  | Non-dihydropyridine calcium channel blockers | 1 | 0.4 |
| **Diuretics** |  | 19 | 8.5 |
|  | Thiazide diuretics | 15 | 6.7 |
|  | Loop diuretics | 7 | 3.1 |
|  | Potassium sparing diuretics | 2 | 0.9 |
| **Cholinesterase inhibitors** |  | 18 | 8.1 |
| **Oral Medications for Diabetes** |  | 18 | 8.1 |
|  | Biguanides | 16 | 7.2 |
|  | Meglitinides | 6 | 2.7 |
|  | Sulfonylureas | 6 | 2.7 |
|  | Sodium glucose transporter (SGLT-2) inhibitors | 4 | 1.8 |
|  | Dipeptidyl peptidase-4 (DPP-4) inhibitors | 1 | 0.4 |
|  | Thiazolidinediones | 1 | 0.4 |
| **Memory Medications** |  | 18 | 8.1 |
| **Angiotensin-2 receptor blockers** |  | 17 | 7.6 |
| **NMDA Receptor Blockers** |  | 16 | 7.2 |
| **Benzodiazepines** |  | 16 | 7.2 |
| **Anticoagulation** |  | 12 | 5.4 |
| **Barbiturates** |  | 11 | 4.9 |
| **Probiotics** |  | 9 | 4.0 |
|  | *Lactobacillus rhamnosus* | 2 | 0.9 |
|  | *Lactobacillus acidophilus* (solo) | 1 | 0.4 |
|  | *Lactobacillus acidophilus* (combo) | 1 | 0.4 |
| **Injectable Diabetes medications** | Glucagon-like peptide-1 receptor agonists (GLP-1RAs) | 8 | 3.6 |
|  | Injectable Insulin Medication | 5 | 2.2 |
| **Atypical Antipsychotics** |  | 5 | 2.2 |
| **Oral corticosteroids** |  | 3 | 1.3 |
| **Immunosuppressive** |  | 3 | 1.3 |
| **Vasodilators** |  | 3 | 1.3 |
| **Carboxamides** |  | 2 | 0.9 |
| **Sulfonamides** |  | 2 | 0.9 |
| **Chemotherapy Medication** |  | 2 | 0.9 |

Supplemental Table 2: Results of linear mixed-effect models for prediction of diversity in GAINS samples

| *Alpha Diversity ~ Sex + Age+ Sample Day+ Education+ Abx Use + Status+ (1\|study id)* | | | | |
| --- | --- | --- | --- | --- |
| Variable | **Value** | **Std Error** | **DF** | **p-value** |
| (Intercept) | 26.1052995 | 10.194995 | 436 | 0.01078497 |
| Sex (male) | 0.35107479 | 1.53567024 | 126 | 0.81954004 |
| Age | -0.1125021 | 0.10697484 | 126 | 0.29496432 |
| Sample Day | 0.00038433 | 0.00142376 | 436 | 0.78733167 |
| Education (years) | 0.11895518 | 0.26083751 | 126 | 0.64913844 |
| Status (Linear contrast) | -0.6677121 | 1.6691115 | 126 | 0.68980413 |
| Status (Quadratic contrast) | -1.9096678 | 1.81606289 | 126 | 0.2950219 |
| Abx usage (past 6months) | -4.3632227 | 1.76756727 | 126 | 0.0149088 |

Supplemental Table 3: All MERF regression model statistics presented as median [M.A.D].

| **Outcome** | **Feature type** | **Population** | **Correlation Coef.** | **RMSE** |
| --- | --- | --- | --- | --- |
| ADAS-Cog-13 | Species Abundance | HC | 0.640 [0.05] | 3.028 [0.19] |
|  |  | MCI | 0.873 [0.04] | 3.574 [0.6] |
|  |  | AD | 0.941 [0.03] | 7.519 [1.1] |
| Memory Z-score | Species Abundance | HC | 0.626 [0.03] | 0.333 [0.01] |
|  |  | MCI | 0.825 [0.07] | 0.394 [0.05] |
|  |  | AD | 0.929 [0.01] | 0.505 [0.06] |
| Executive Function Z-score | Species Abundance | HC | 0.718 [0.02] | 0.399 [0.06] |
|  |  | MCI | 0.852 [0.04] | 0.446 [0.06] |
|  |  | AD | 0.846 [0.04] | 1.569 [0.1] |
| ADAS-Cog-13 | Metabolic Pathways | HC | 0.627 [0.06] | 2.995 [0.2] |
|  |  | MCI | 0.874 [0.04] | 3.508 [0.7] |
|  |  | AD | 0.940 [0.02] | 6.493 [1.9] |
| Memory Z-score | Metabolic Pathways | HC | 0.605 [0.02] | 0.325 [0.03] |
|  |  | MCI | 0.813 [0.07] | 0.388 [0.04] |
|  |  | AD | 0.944 [0.02] | 0.444 [0.08] |
| Executive Function Z-score | Metabolic Pathways | HC | 0.720 [0.02] | 0.400 [0.04] |
|  |  | MCI | 0.806 [0.04] | 0.483 [0.07] |
|  |  | AD | 0.882 [0.04] | 1.312 [0.2] |
| ADAS-Cog-13 | KO Terms | HC | 0.611 [0.04] | 3.105 [0.1] |
|  |  | MCI | 0.876 [0.03] | 3.323 [0.5] |
|  |  | AD | 0.935 [0.03] | 6.279 [1.5] |
| Memory Z-score | KO Terms | HC | 0.629 [0.03] | 0.322 [0.03] |
|  |  | MCI | 0.805 [0.06] | 0.394 [0.02] |
|  |  | AD | 0.953 [0.01] | 0.474 [0.04] |
| Executive Function Z-score | KO Terms | HC | 0.714 [0.03] | 0.414 [0.04] |
|  |  | MCI | 0.795 [0.05] | 0.455 [0.04] |
|  |  | AD | 0.873 [0.04] | 1.461 [0.2] |
